# Violence in childhood and community contexts: a multi-level model of factors associated with women's intimate partner violence experience in Samoa

**DOI:** 10.1016/j.lanwpc.2023.100957

**Published:** 2023-11-18

**Authors:** Hattie Lowe, Jenevieve Mannell, Taiaopo Faumuina, Lewis Sinclair, Lineta Tamanikaiyaroi, Laura Brown

**Affiliations:** a Institute for Global Health, University College London, 30 Guilford Street, London, WC1N 1EH, UK; b Samoa Bureau of Statistics, Apia, P.O. Box 1151, Samoa; c Faculty of Technical Education, National University of Samoa, Apia, P.O. Box 5768, Samoa

**Keywords:** Intimate partner violence, Cross-sectional studies, Community-based interventions, Samoa

## Abstract

**Background:**

Intimate partner violence (IPV) affects approximately 26% of women worldwide and is driven by a complex interplay of factors across individual, relationship/household, community and societal levels. Individual and relationship/household factors are well studied however little empirical evidence exists on factors at the community level that drive IPV which are needed to inform prevention interventions.

**Methods:**

We conducted a cross-sectional, multi-level analysis of factors associated with women's IPV experience in Samoa using the 2019–20 Demographic and Health Multiple Indicator Cluster Survey. We used hierarchical multivariable logistic regression to assess individual, relationship/household and community level effects on women's risk of physical, sexual and/or emotional IPV.

**Findings:**

The past year prevalence of physical, sexual and/or emotional IPV among women in Samoa was 31.4%. At the individual and relationship/household level, women's employment, witnessing IPV between parents, experiencing physical abuse from a parent, and partner's alcohol use and controlling behaviours were associated with higher risk of IPV. At the community level, higher levels of women with higher education and involved in household decision-making, and higher levels of men in employment were protective against IPV.

**Interpretation:**

A complex interplay of factors across individual, relationship/household and community levels are associated with women's experience of IPV in Samoa. Experiences of IPV are embedded within a broader context of violence against children and harmful alcohol use. Community contexts, including women's empowerment and men's employment, are also associated with women's IPV experience in Samoa. These findings not only demonstrate that public health issues such as IPV, violence against children and harmful alcohol use should be addressed together as part of multi-pronged approaches, but they point towards the importance of community-level analyses for designing and delivering community-based interventions. Greater knowledge of community dynamics will enable community-based interventions to create environments at the community level that support meaningful and sustainable change towards IPV prevention.

**Funding:**

Funding for this study was provided by UKRI (ref. MR/S033629/1).

## Translated abstract

**Oto’otoga o le Sue’suega:**

E tusa ma le 26 % o tamaitai ua aafia I saua’ga i faigapa’aga i le lalolagi atoa e afua mai i ni mafua’aga mai le tagata lava ia, fai’a ma le aiga o loo nofotane ai, faapea lona tulaga I lona i lona nuu po o le sio’siomaga o nofo ai. O faigapaa’ga taitoatasi ma le siosiomaga I aiga taitasi o nonofo ai sa mafuli I ai lenei sue’suega, ae e faa’leai ni mafuaaga o sau’aga i nuu e manaomia e fuafua ai ni tali o lenei faa’fitauli tu’ga.

**Auala na Faa’ogaina I le Suesuega:**

Sa faa’ogaina ni i’uga o mafuaaga o Sau’aga o Faigapaaga mai le Nofoaga filiafila e le suesuega a le Soifua Maloloina na aa’fia ai tamaitai Samoa mai le tausaga 2019-20. Sa faaogaina le metotia ua taua faaperetania (Hierarchical Multivariable Logistic Regression) e suesueina ai tama’itai taitasi ua aa’fia ma latou fai’a ma aiga o latou paaga, o le aa’fiaga o nuu ma nofoaga i nei ituaiga sau’aga.

**Tau’nuuga o le Suesuega:**

E tusa ma le 31.4% o tamaitai Samoa na aa’fia i sauaga faa’faigapaaga e aafia ai le tino, feusuaiga, mafaufau ma lagona. O le va o le tamaitai ma le aiga o lana paaga I le itu I le tamaoaiga, lona tulaga I galuega ua molimauina ai sau’aga I le va o matua o le tamaitai ma matua foi o lana pa’aga. O upu faa’luma’luma a matua, o le taumafa ava malosi ma le pule’pule tutu o matua I fanau o tulaga ia ua avea ma faapogai o sau’aga I le va o tamaitai ma latou paa’ga. I totonu o nuu ma alalafaga e maualuga le numera o tamaitai aoaoina lelei o oloo auai I le faiga o faaiuga faapea foi ta’malii faigaluega lelei latou te puipui’a tama’itai mai sauaga ma latou paa’ga.

**Au’iliiliga o le Sue’su’ega:**

E lavelave ma faigata ona manino mai mafua’ga e aa’fia ai tamaitai i sauaga fai’faapaaga ona o le mafua’aga e mai lava i a te ia ma lona va ma lona aiga o loo nofotane ai, o matua aemaise foi le nuu. O aa’fiaga o nei sa’uaga ua aafia ai le fanau ona o le inu ava malosi le tau’pulea. O le siitia o tulaga o le aoaoina ma le tamaoaiga o tina ma le lelei o galuega a alii i nuu ma a’lala’faga, ua maitauina ua avea ma mafuaa’ga o nei sau’aga i Samoa. O tau’nuuga o lenei suesuega, ua faa’ilo ai i le vaega o le Soifua Maloloina Lautele o Sau’aga o fanau ma le inu ava malosi le tau pule’a e ta’tau ona faavae ai ni fofo o lenei faafitauli o Sau’aga o faa’faigapaaga e taulamua ai nuu ma alalafaga. O le ao’ga o le malamalama lelei I faavae o nuu ma alalafaga, o se atamai sili lelei ona lelei e faataatiatia ai alafua e gafataulimaina e nuu taitasi ina ia faa’foia ai Sau’aga I Fai’gapaaga mo se nofo lelei o aiga taitasi.

**Faatupeina o lenei Su’esu’ega:**

O lenei Suesuega na faa’tupeina e le faa’lapotopotoga ale UKRI (ref. MR/S033629/1).

**Disclaimer:**

This translation in Samoan was submitted by the authors and we reproduce it as supplied. It has not been peer reviewed. Our editorial processes have only been applied to the original abstract in English, which should serve as reference for this manuscript.

**Research in context**

**Evidence before this study**

Factors associated with intimate partner violence (IPV) experiences at the individual and relationship/household level have been well documented in the literature. Women's own characteristics (including age, education, disability status, socioeconomic status, employment and experiences of other forms violence), as well as her partner's characteristics (including age, education, employment and substance abuse) contribute to her risk of experiencing IPV. However, little empirical evidence exists on factors associated with IPV at the community level. The small but growing evidence base suggests that area-level education, poverty, and social norms might have a role to play, but more research is needed. Despite facing one of the highest IPV regional prevalence rates globally, IPV in the Pacific region is vastly under researched.

**Added value of this study**

Our results at the individual and relationship/household level support findings from other contexts on factors associated with IPV, namely that women's employment, childhood exposure to and experience of violence, and partner's alcohol use and controlling behaviours are associated with IPV experience. Our study adds to a growing evidence base on community factors, demonstrating that in communities with higher levels of women with higher education, women involved in household decision-making and men in paid employment, women have a lower risk of experiencing IPV. Together, these findings make an important and timely contribution to the literature on the factors associated with IPV in Samoa and the Pacific Islands region, as a vastly under researched area.

**Implications of all the available evidence**

There are similarities across contexts on factors associated with IPV at the individual and relationship/household level. This study is the first in the Pacific Islands region to provide evidence of the factors associated with IPV at the community level. We argue that it is essential to understand community contexts for the development of community-based interventions to prevent IPV in the Pacific and beyond. Further research is needed to better measure key community-level factors and develop understandings of how they contribute to high levels of IPV.

## Introduction

Violence against women and girls (VAWG) is a major public health crisis and human rights violation, with an estimated 26% of women experiencing physical and/or sexual intimate partner violence (IPV) in their lifetime,^1^ the most common form of VAWG. IPV is defined as “any behaviour by a current or former male intimate partner that causes physical, sexual or psychological harm”.^1^ IPV presents a major health, social and economic burden to women, their families and societies, requiring urgent and sustained global attention and funding to eliminate it.^1^, ^2^, ^3^

IPV is understood to be driven by multiple factors operating at different levels of the ecological framework,^4^ including the individual, community and societal levels.^4^, ^5^, ^6^, ^7^, ^8^, ^9^ In the global literature, most studies focus on the individual level, finding that a woman's own characteristics, such as age, education, disability status, socioeconomic status, employment and exposure to other forms of violence, contribute to her risk of IPV,^5^^,^^6^^,^^8^ as well as factors pertaining to her partner, such as his age, education, and substance use.^5^^,^^6^^,^^8^^,^^10^ Factors driving IPV at the community level have been less well studied, with early evidence predominantly focusing on high-income settings.^11^ Studies in the USA found that in communities with higher social cohesion and collective efficacy, women were at lower risk of experiencing IPV, while in communities with higher levels of socioeconomic deprivation, women were at increased risk of experiencing IPV.^11^ There is a small but growing evidence base suggesting that area level (community, district and regional) factors also play an important role in increasing a woman's risk of IPV in low- and middle-income countries (LMICs), including area-level education, poverty, and social and gender norms.^6^^,^^11^^,^^12^ More recent global evidence points towards intersecting structural factors, including conflict, colonialism, patriarchy, and climate change, in creating high risk contexts for IPV perpetration and experience.^13^

Understanding community contexts is important for intervention development, particularly given the move over the past decade towards implementing community-based interventions for IPV prevention in LMICs, and the knowledge that communities play a pivotal role in responding to and reducing violence.^14^, ^15^, ^16^ While to the best of our knowledge no such studies have been conducted in the Pacific region, there is a small but growing evidence base emerging across LMICs. Nationally-representative data from Tanzania found that a higher acceptance of wife beating among women at the community level was associated with increased risk of experiencing IPV,^12^ consistent with findings from Bangladesh,^17^ the Democratic Republic of the Congo,^18^ and Nigeria.^19^ Theoretically, it is proposed that living in a community with a collective tolerance for VAWG upholds an environment of male authority and enables VAWG.^12^ Area-level poverty is another community-level driver of IPV risk.^6^ In Uganda, increasing regional levels of women in the poorest households was associated with higher levels of IPV,^20^ as was higher levels of male unemployment in Tanzania.^12^ Area-level deprivation is hypothesised to exacerbate other risk factors for IPV, such as substance abuse and household conflict.^6^ A small number of studies found protective associations with increasing women's education, hypothesising that education levels can reduce IPV risk through access to economic opportunities, increased bargaining power and opportunities to leave violent relationships.^18^^,^^21^ However, conflicting evidence finds that higher levels of men's education in the community leads to women's increased risk of IPV, demonstrating that the relationship between community risk factors and IPV is complex and still not well understood.^12^

Despite this growing body of evidence, more multi-level analyses that explore factors associated with IPV at the individual, relationship/household and community level simultaneously are needed to build consensus and theory on how individual and community contexts influence IPV experience and perpetration.^6^ Towards this aim, we conducted a cross-sectional, multi-level study of the factors associated with IPV in Samoa as part of the EVE (Evidence for Violence Prevention in the Extreme) Project, which is developing the evidence for how to prevent VAWG in the world's highest prevalence settings.^22^ The research questions used to inform our analysis were: (1) what are the community-level factors that are associated with IPV experience for women in Samoa? (2) Taking individual and relationship factors into account, to what extent do community-level factors explain IPV risk? Multi-level analysis was chosen to answer these research questions for its ability to investigate individual experiences using information from data aggregated at higher hierarchal levels (i.e., the community).

### Violence against women and girls in Samoa

Women and girls in Samoa, an independent state located in the South Pacific Ocean, face one of the highest prevalence rates of IPV globally, with 39.6% of women reporting having experienced physical, sexual and/or emotional violence from an intimate partner in their lifetime, according to the Samoa 2019–20 DHS-MICS (Demographic and Health Multiple Indicator Cluster Survey)^d^.^23^ Samoa has a population size of approximately 200,000 inhabitants living across two main islands, Upolu, where the capital Apia is located, and Savai'i, a larger more rural island. Samoa has a predominantly Christian population and social and cultural life is heavily influenced by the Christian church and the *Fa'a Samoa*, an indigenous way of life dating back more than 3000 years. *Fa'a Samoa* consists of values and traditions that guide the way of life in Samoa centred around the *aiga* (family) and the *fa'amatai* (chiefly governance system).

IPV in Samoa is embedded within a broader epidemic of family violence, believed to be driven by complex and inter-related factors such as intergenerational violence, harmful physical and psychological punishment of children, gender norms and inequalities, low levels of women's empowerment, and misinterpretations of the *Fa'a Samoa* and the Bible.^24^ The 2013 Family Safety Act is key piece of legislation in Samoa introduced to “provide for greater protection of families and the handling of domestic violence and related matters” including the provision of protection orders for survivors.^24^ The Samoa Victim Support Group (SVSG), a non-governmental organisation, is the main service provider for survivors of violence in Samoa with a national helpline and the only women's and children's shelter. SVSG implement a range of tertiary prevention activities across Samoan communities and have a network of village representatives to help identify vulnerable individuals in their communities. There have also been a number of government programmes established by relevant ministries, including the Ministry of Women, Communities and Social Development (MWCSD), and the Ministry of Police, including public education programmes and the establishment of a network of women village representatives to act as a liaison between the women's committee in the villages and the MWCSD. The growing awareness of family violence and VAWG in Samoa and the publication of reports such as the National Inquiry into Family Violence by the Office of the Ombudsman^24^ highlight the urgent need to gain a more comprehensive understanding of the drivers of the high prevalence of violence in this context in order to implement relevant and sustainable solutions.

## Methods

In this cross-sectional study of factors associated with women's experience of IPV, we analysed nationally representative data from the 2019 to 20 Samoa Demographic and Health Multiple Indicator Cluster Survey (DHS-MICS).^23^ The DHS-MICS employed a multi-stage, stratified cluster sampling approach based on the 2016 Samoa Census of Population and Housing, stratified by urban or rural area, and the four regions (Apia Urban Area, North West Upolu, Rest of Upolu, and Savai'i).^23^ The four statistical regions are comprised of 51 political districts, 339 villages, and 668 enumeration areas, which comprise groupings of households. In the first of the two stages, 204 clusters (primary sampling units) were selected from 668 total clusters based on the 2016 census enumeration areas. In the second stage, a list of households in each enumeration area was created, and 20 households in each rural enumeration area and 15 households in each urban enumeration area were selected using a random systematic selection procedure. In total, 3675 households were randomly selected for interview, including 1215 urban households and 2460 rural households.

The Samoa DHS-MICS implemented seven questionnaires, of which data from three were extracted for this particular study: 1) a women's questionnaire administered to all women aged 15–49 years in every household (unweighted n = 4,139, response rate 91.4%), 2) a domestic violence questionnaire administered to one randomly selected woman aged 15–49 years in each of the households where women were selected (unweighted n = 2130) and 3) a men's questionnaire administered to all men aged 15–49 years in every third household (unweighted n = 1204, response rate 82.1%). The domestic violence questions used in this analysis employed the DHS domestic violence questionnaire methodology.

This study draws on the integrated ecological framework^4^ to explore the factors associated with women's IPV experience in Samoa across individual, relationship/household and community levels, with a particular focus on the community-level as an important gap in the literature. We used a combination of theoretical and empirical reasoning to select which variables to include in our analysis. Some factors, such as age, education and employment, were determined as important to include a priori, as these are well-established as being associated with IPV in the literature.^5^^,^^6^ A more exploratory approach was used with the remaining variables as their relationships are less well established, drawing upon our contextual knowledge, three reviews of the literature on community-level factors associated with IPV in other contexts,^5^^,^^6^^,^^11^ and the bivariate analyses performed in stage one of data analysis. We used the ecological levels from the integrated ecological framework to group the explanatory variables in our analysis, and these groupings were also used to build our logistic regression models.

### Variables

#### Outcome variable: current experience of intimate partner violence

The outcome variable was derived from the domestic violence questionnaire. Ever-partnered women, defined as women who had ever been married or cohabited with a partner, were asked 13 questions related to emotional, physical and sexual violence perpetrated by a current or former partner (Table 1). These questions were coded as yes or no, and women who answered yes were asked whether the violence occurred *often, sometimes* or *not at all* in the past 12 months. Current experience of partner violence was defined as a woman experiencing one or more acts of physical, sexual or emotional violence *often* or *sometimes* in the past 12 months. Past 12-month experience of IPV was selected over lifetime experience as the outcome of interest in this study because of its greater stability^25^ and relevance to this analysis which attempts to explore factors associated with IPV experience. While we cannot determine temporality of events using cross-sectional data, using past 12-month experience of IPV is more likely to produce a logical sequencing of events (explanatory factors before IPV experience) than lifetime prevalence. There was no missing data for any of the IPV items; all eligible women (ever-partnered women) responded to all items (missingness = 0%).

**Table 1:** Coding of newly derived variables (variables created by combining or recoding original variables).

Measure	Concept	Question(s)	Coding
**Outcome variable**			
Current experience of IPV	Emotional IPV	Did your (last) (husband/partner) ever: a) say or do something to humiliate you in front of others? b) Threaten to hurt or harm you or someone you care about? c) Insult you or make you feel bad about yourself?	Yes (1), no (0) Women who answered yes were asked whether the violence occurred *often (1), sometimes (2)* or *not at all (0)* in the past 12 months. Current experience defined as experiencing one or more acts of physical, sexual or emotional violence often or sometimes in the past 12 months
	Physical IPV	Did your (last) (husband/partner) ever: d) push you, shake you, or throw something at you? e) Slap you? f) Twist your arm or pull your hair? g) Punch you with his fist or with something that could hurt you? h) Kick you, drag you, or beat you up? i) Try to choke you or burn you on purpose? j) Threaten to attack you with a knife, something sharp, or another weapon?	
	Sexual IPV	Did your (last) (husband/partner) ever: k) physically force you to have sexual intercourse with him when you did not want to? l) Physically force you to perform any other sexual acts you did not want to? m) Force you with threats or in any other way to perform sexual acts you did not want to?	
**Explanatory variables**			
Justification of wife beating	Women's attitudes towards wife beating	In your opinion, is a husband justified in hitting or beating his wife in the following situations: a) if she goes out without telling him? b) If she neglects the children? c) If she argues with him? d) If she refuses to have sex with him? e) If she burns the food? f) If she comes home late?	(1) Yes—if respondent said yes to one or more circumstances (0) No—If respondent said no to all circumstances
	Men's attitudes towards wife beating		
Physical abuse by parents	Non-partner physical violence	1) From the time you were 15 years old has anyone other than (your/any) (husband/partner) hit you, slapped you, kicked you, or done anything else to hurt you physically? 2) Who has hurt you in this way?	Physical abuse by parents was coded as Yes (1) if woman answered yes to Q1 and then answered mother/step-mother or father/step-father to Q2. Coded as no if woman answered No (0) to Q1.
Women's involvement in decision making	Household decision making	Who usually makes decisions about: a) health care for yourself? b) Major household purchases? c) Purchases of daily household needs? d) Visits to your family or relatives? e) How your husband's/partner's earnings will be used?	Coded as Yes (1) if the woman responded ‘you’ or ‘you and your husband/partner’ across all decision-making domains. Coded as No (0) if woman responded ‘husband/partner’ or ‘someone else’ across any decision-making domains.
Relationship control	Partner's controlling behaviours	Please tell me if these apply to your relationship with your (current/last) (husband/partner): a) he is jealous or angry if you talk to another man? b) He frequently accuses you of being unfaithful? c) He does not permit you to meet your female friends? d) He tries to limit your contact with your family? e) He insists o knowing where you are at all times? f) He does not allow you to join any social functions?	Coded as Yes (1) if woman answered yes to any of the controlling behaviour questions a-f. Coded as No (0) if woman answered no to all controlling behaviour questions a-f.

#### Explanatory variables

##### Individual and relationship/household level characteristics

Data pertaining to women's individual, relationship and household characteristics were derived from the individual women's and domestic violence questionnaires. Sociodemographic characteristics included age, education, marital status, employment, and disability status. Factors pertaining to the individual woman and her experiences included her attitudes towards physical VAW, whether she had a father who had beaten her mother, whether she experienced physical abuse from her mother/step-mother or father/step-father since the age of 15, and whether she had experienced non-partner sexual violence in her lifetime.

Characteristics of the woman's household included region and household wealth quintile. Factors pertaining to the woman's relationship with her partner included partner's age, partner's alcohol use, partner's controlling behaviour and whether she is involved in making all household decisions with her partner jointly or on her own (Table 1).

##### Community level characteristics

To generate community-level variables, we aggregated individual responses for each item at the cluster (community) level. Clusters represent enumeration areas from the 2016 census, made up of groupings of households. Aggregated data at the cluster level were then grouped into tertiles, to provide estimates of high, medium and low levels of the variable in the community to enable comparison. Data pertaining to women's characteristics at the community level were derived from the women's survey, and data pertaining to men's characteristics at the community level were derived from the men's survey. We explored community levels of women: in the wealthiest quintile (i.e., communities with low, medium and high levels of women in the richest wealth quintile), with post-secondary education, in cash employment, who never justify physical IPV, involved in decision making, with experience of parental physical abuse, and with experience of non-partner sexual violence. We also explored community levels of men with higher education, in cash employment, and who never justify physical IPV.

### Analysis

Data analysis was performed using STATA17. We first used Pearson's chi-squared test to assess the bivariate association between the explanatory variables and the outcome of interest, past 12-month experience of physical, sexual and/or emotional IPV. The results of this analysis were used to inform which variables would be added to the logistic regression model in stage two of our analysis, based on a significance level of Bonferroni-adjusted alpha of 0.0019 to mitigate against the Type I error inherent in multiple comparisons. Some factors, such as age, experiences of non-partner sexual violence and attitudes towards IPV, were included in the model despite their associations with IPV being outside of this level of significance due to being a-priori selected for inclusion in the model based on strong indication from the global literature on risk factors for IPV.^5^^,^^6^ Community-level factors were also included in the model after being a-priori selected for inclusion based on their relevance to the research questions under study.

In stage two of the analysis, we used unadjusted logistic regression to assess the crude associations between the exposure variables and the outcome of interest. Associations were then assessed in a hierarchical, multivariate logistic regression model (with individual women set as level one and communities (clusters) set as level two, i.e., women are nested within communities). We used mixed effects logistic regression models with the *melogit* command in Stata as the outcome of interest (IPV) was binary. The conditional distribution of the outcome given the random effects is assumed to be Bernoulli, with success probability determined by the logistic cumulative distribution function. We conducted two-level logistic regression models with random intercepts at the community level. No random coefficients were added as conceptually none of our variables were thought to have a varying effect on IPV across communities. As we had no random coefficients, we used the default variance covariance structure (i.e., there was no need to allow for the random effects to be correlated, as there weren't any). Model 1 assessed the relationship between women's individual level characteristics and the outcome of interest, model 2 included individual and relationship/household characteristics, and model 3 contained all variables, including the individual, relationship/household, and community-level characteristics. All analyses were weighted using Samoa DHS-MICS 2019–20 sample weights to account for the stratified clustered sampling design.

## Results

The prevalence of current physical, sexual and/or emotional IPV was 31.4% among ever-married or partnered women (95% CI 28.1–34.9). The majority of women (79.9%) lived on Upolu Island and were married or in union (63.4%) (Table 2). Almost all women had secondary (70.5%) or higher education (27.4%), and a large percentage of women were unemployed (63.4%).

**Table 2:** Demographic characteristics of women in the domestic violence module.

Characteristic	Percentage	Number of women
Total	100.00	3667
**Age**		
15–19	17.9	656
20–24	17.8	654
25–29	14.6	535
30–34	12.4	455
35–39	11.6	425
40–44	12.8	470
45–49	12.9	472
**Employment status**		
Work for pay/profit	19.9	728
Unpaid work	1.4	52
Unemployed	63.4	2325
Student	14.7	540
Incapable/other/missing	0.6	22
**Education**		
Primary	2.1	77
Secondary	70.5	2567
Higher	27.4	1000
**Marital status**		
Never married	33.9	1242
Married or in union	63.4	2319
Divorced	2.7	98
**Region**		
Island of Upolu		
Apia Urban Area	21.3	781
North West Upolu	35.0	1281
Rest of Upolu	22.8	836
Island of Savai'i	20.1	768
**Wealth**		
Poorest quintile	18.4	675
Second	19.7	721
Middle	20.2	741
Fourth	20.5	750
Richest quintile	21.2	779
**Disability**		
Some functional difficulty	1.3	41
No functional disability	98.7	3206

### Bivariate analysis

The bivariate analyses illustrated significant differences in the prevalence of current physical, sexual and/or emotional IPV experience among women by individual, relationship/household and community level characteristics (Table 3). At the individual level, IPV was significantly more prevalent among women who had primary (33.6%) or secondary education (34.3%), who were married or cohabiting (32.2%), who were unemployed (34.4%) or students (35.0%), and who reported childhood exposure to violence (witnessing their father beat their mother (41.5%) and experiencing physical abuse themselves from their mother/step-mother or father/step-father (40.1)). At the household level, IPV was significantly more prevalent among women in the rural region of North West Upolu (37.6%) and women in the poorer wealth quintiles (poorest 38.7%, second poorest 34.2%). In relationships, IPV was significantly more prevalent for women who reported their partner drinking alcohol (39.8%), being excluded from certain household decisions (37.7%), and experiencing controlling behaviour from their partner (38.2%). Finally, at the community level, IPV was significantly less prevalent among women living in communities with higher levels of wealth (23.4%), higher levels of women with post-secondary education (22.5%), lower levels of parental IPV (26.7%), and higher levels of men in cash employment (26.2%).

**Table 3:** Prevalence of current physical, sexual and/or emotional intimate partner violence among ever-married or partnered women by women's and community characteristics.

Characteristic	Physical, sexual and or emotional IPV (%)	p-value^a^
Total	31.4	
**Women’s characteristics**		
**Age**		
15–19^b^	31.6	0.06
20–24	45.9	
25–29	27.1	
30–34	32.6	
35–39	30.2	
40–44	30.7	
45–49	26.9	
**Education**		
Primary	33.6	0.00
Secondary	34.3	
Higher	22.1	
**Marital status**		
Married or living together	32.2	0.00
Divorced/widowed/separated	13.1	
**Employment**		
Work for pay/profit	21.9	0.00
Unpaid work	8.3	
Unemployed	34.4	
Student	35.0	
Incapable/other/missing	23.2	
**Disability**		
At least one type of functional difficulty	31.1	0.98
No functional difficulty	31.4	
**Woman's attitudes towards wife beating**		
Justified wife beating in one or more circumstances	34.3	0.15
No justification	29.3	
**Woman's father beat mother**		
Yes	41.5	0.00
No	27.5	
Don't know	28.7	
**Woman experienced physical abuse from mother or father since age 15**		
Yes	40.1	0.00
No	20.1	
**Woman experienced non-partner sexual violence in her lifetime**		
Yes	44.4	0.01
No	29.8	
**Relationship/Household characteristics**		
**Region**		
Apia Urban Area	28.0	0.02
North West Upolu	37.6	
Rest of Upolu	30.5	
Savai'i	24.6	
**Wealth**		
Poorest	38.7	0.00
Second	34.2	
Middle	30.5	
Fourth	32.8	
Richest	20.3	
**Partner's age**		
18–30	37.5	0.23
31–40	29.4	
41–50	30.7	
51–76	33.6	
**Partner drinks alcohol**		
Yes	39.8	0.00
No	23.8	
**Woman is involved in making all household decisions with her partner or on her own**		
Yes	30.0	0.04
No	37.7	
**Woman experiences controlling behaviour from her partner**		
Yes	38.2	0.00
No	7.1	
**Community characteristics**		
**Community level of women in wealthiest quintile**		
Low	33.7	0.04
Medium	33.6	
High	23.4	
**Community level of women with post-secondary education**		
Low	35.3	0.02
Medium	32.6	
High	22.5	
**Community level of women in cash employment**		
Low	26.7	0.37
Medium	32.8	
High	32.6	
**Community level of women with no reasons to justify wife beating**		
Low	30.8	0.37
Medium	35.0	
High	28.8	
**Community level of women who are involved in all decision making**		
Low	38.0	0.01
Medium	25.7	
High	29.3	
**Community level of women whose father didn't beat their mother**		
Low	36.4	0.05
Medium	29.8	
High	26.7	
**Community level of women who experienced physical abuse from their parents since age 15**		
Low	27.2	0.14
Medium	31.1	
High	36.8	
**Community level of women who experienced non-partner sexual violence in their lifetime**		
Low	29.0	0.68
Medium	32.5	
High	32.2	
**Community level of men with higher education**		
Low	29.8	0.75
Medium	32.0	
High	32.8	
**Community level of men in cash employment**		
Low	36.6	0.04
Medium	32.0	
High	26.2	
**Community level of men who never justify IPV**		
Low	32.1	0.93
Medium	30.3	
High	31.6	

^NB^All estimates are derived from the responses of 2417 women other than for education (n = 2408), disability (n = 2407), partner's age (n = 2317), partner's alcohol use (n = 2368) and decision-making (n = 2319).

a Bonferroni-adjusted alpha = 0.0019 (i.e., 0.05 divided by 26).

b Figures derived from weighted sample of only 39 individuals.

### Hierarchical multivariate logistic regression analysis of factors associated with IPV

Table 4 presents the results from the hierarchical multivariate logistic regression. Model 1 includes factors relating to women's individual characteristics. Compared with women aged 45–49, women aged 20–24 were at significantly higher risk of experiencing IPV (OR 2.25; CI 1.22–4.11), as were women with secondary education (OR 1.63; CI 1.09–2.44) when compared to women with higher education. Working for pay (OR 0.58; CI 0.38–0.90), as well as unpaid work (OR 0.20; CI 0.51–0.82), demonstrated a statistically significant protective association with IPV when compared to being unemployed. Reporting that her father beat her mother (OR 1.91; CI 1.30–2.81), experiencing physical violence from her mother or father (OR 2.11; CI 1.49–2.97), and experiencing non-partner sexual violence (OR 1.78; CI 1.02–3.09), significantly increased a woman's risk of experiencing IPV when compared to no experience of these types of violence.

**Table 4:** Hierarchical multivariate logistic regression models of factors associated with current physical, sexual and/or emotional IPV for ever-partnered women.

Characteristic	Unadjusted model			Model 1 (individual)			Model 2 (relationship/household)			Model 3 (community)		
	OR (95% CI)	p-value	Wald test p-value	OR (95% CI)	p-value	Wald test p-value	OR (95% CI)	p-value	Wald test p-value	OR (95% CI)	p-value	Wald test p-value
**Individual**												
**Age**												
15–19^a^	1.11 (0.25–4.99)	0.90	0.16	0.96 (0.22–4.21)	0.96	0.14	1.02 (0.24–4.29)	0.98	0.35	1.10 (0.26–4.64)	0.89	0.33
20–24	2.23 (1.22–4.09)	0.01		2.25 (1.22–4.11)	0.00		1.79 (0.91–3.48)	0.09		1.80 (0.93–3.47)	0.08	
25–29	1.06 (0.60–1.87)	0.83		1.25 (0.72–2.18)	0.42		0.94 (0.50–1.78)	0.85		0.95 (0.50–1.80)	0.87	
30–34	1.39 (0.79–2.46)	0.25		1.54 (0.89–2.69)	0.12		1.24 (0.66–2.35)	0.50		1.25 (0.66–2.37)	0.48	
35–39	1.15 (0.64–2.04)	0.64		1.13 (0.65–1.98)	0.66		0.88 (0.48–1.63)	0.69		0.88 (0.48–1.64)	0.70	
40–44	1.38 (0.76–2.52)	0.29		1.35 (0.73–2.48)	0.34		1.14 (0.58–2.25)	0.70		1.16 (0.59–2.26)	0.67	
45–49	ref	ref		ref	ref		ref	ref		ref	ref	
**Education**												
Primary	1.52 (0.59–3.89)	0.38	0.01	1.50 (0.57–3.94)	0.42	0.06	1.50 (0.60–3.77)	0.38	0.27	1.43 (0.58–3.52)	0.44	0.37
Secondary	1.77 (1.21–2.59)	0.00		1.63 (1.09–2.44)	0.02		1.41 (0.93–2.15)	0.11		1.35 (0.89–2.07)	0.16	
Higher	ref	ref		ref	ref		ref	ref		ref	ref	
**Employment status**												
Unemployed	ref	ref	0.00	ref	ref	0.03	ref	ref	0.03	ref	ref	0.03
Work for pay	0.48 (0.32–0.72)	0.00		0.58 (0.38–0.90)	0.02		0.59 (0.36–0.96)	0.03		0.59 (0.36–0.97)	0.04	
Unpaid work	0.18 (0.04–0.71)	0.01		0.20 (0.51–0.82)	0.03		0.14 (0.03–0.59)	0.00		0.13 (0.03–0.58)	0.00	
Student	1.40 (0.34–5.72)	0.64		1.55 (0.32–7.47)	0.58		1.34 (0.28–6.5)	0.71		1.42 (0.29–6.97)	0.66	
**Parental IPV (father beat mother)**												
No	ref	ref	0.00	ref	ref	0.00	ref	ref	0.01	ref	ref	0.01
Yes	1.89 (1.32–2.70)	0.00		1.91 (1.30–2.81)	0.00		1.92 (1.27–2.92)	0.00		1.91 (1.27–2.89)	0.43	
Don't know	1.40 (0.58–3.39)	0.46		1.73 (0.70–4.24)	0.23		1.48 (0.59–3.75)	0.40		1.46 (0.57–3.69)	0.00	
**Experienced physical abuse from parents since age 15**												
No	ref	ref	0.00	ref	ref	0.00	ref	ref	0.00	ref	ref	0.00
Yes	2.22 (1.56–3.14)	0.00		2.11 (1.49–2.97)	0.00		2.17 (1.49–3.16)	0.00		2.19 (1.51–3.19)	0.00	
**Experienced non-partner sexual violence in lifetime**												
No	ref	ref	0.02	ref	ref	0.04	ref	ref	0.25	ref	ref	0.24
Yes	2.00 (1.14–3.53)	0.02		1.78 (1.02–3.09)	0.04		1.39 (0.80–2.43)	0.25		1.39 (0.80–2.41)	0.24	
**Relationship/household**												
**Wealth**												
Poorest	2.05 (1.09–3.86)	0.03	0.13				1.20 (0.60–2.37)	0.61	0.34	1.11 (0.54–2.30)	0.77	0.28
Second	1.41 (0.79–2.53)	0.24					0.90 (0.47–1.72)	0.74		0.83 (0.41–1.65)	0.59	
Middle	1.57 (0.93–2.64)	0.09					0.90 (0.51–1.54)	0.67		0.82 (0.46–1.45)	0.49	
Fourth	1.97 (1.15–3.38)	0.01					1.45 (0.79–2.67)	0.23		1.38 (0.74–2.57)	0.31	
Richest	ref	ref					ref	ref		ref	ref	
**Current or most recent partner drinks alcohol**												
No	ref	ref	0.00				ref	ref	0.00	ref	ref	0.00
Yes	2.49 (1.79–3.48)	0.00					2.67 (1.93–3.70)	0.00		2.69 (1.94–3.72)	0.00	
**Woman involved in making all family/household decisions (either alone or with partner)**												
No	ref	ref	0.12				ref	ref	0.13	ref	ref	0.17
Yes	0.74 (0.50–1.08)	0.12					0.76 (0.53–1.08)	0.13		0.77 (0.53–1.11)	0.17	
**Woman experienced any controlling behaviours from current partner**												
No	ref	ref	0.00				ref	ref	0.00	ref	ref	0.00
Yes	8.81 (4.97–15.62)	0.00					7.72 (3.88–15.36)	0.00		7.52 (3.78–14.9)	0.00	
**Community**												
**Community level of women in the wealthiest quintile**												
Low	ref	ref	0.04							ref	ref	0.76
Medium	1.01 (0.67–1.54)	0.93								1.04 (0.67–1.62)	0.87	
High	0.60 (0.39–0.92)	0.02								0.82 (0.42–1.59)	0.55	
**Community level of women in higher education**												
Low	ref	ref	0.00							ref	ref	0.09
Medium	1.03 (0.69–1.53)	0.90								0.92 (0.56–1.50)	0.73	
High	0.49 (0.32–0.77)	0.00								0.49 (0.25–0.96)	0.04	
**Community level of women in cash employment**												
Low	ref	ref	0.22							ref	ref	0.56
Medium	1.50 (0.94–2.39)	0.09								0.74 (0.42–1.31)	0.31	
High	1.34 (0.85–2.12)	0.20								0.72 (0.37–1.40)	0.34	
**Community level of women involved in decision making**												
Low	ref	ref	0.00							ref	ref	0.06
Medium	0.2 (0.34–0.79)	0.00								0.62 (0.39–1.00)	0.05	
High	0.63 (0.41–0.96)	0.03								1.08 (0.67–1.73)	0.75	
**Community level of women who never justify IPV**												
Low	ref	ref	0.21							ref	ref	0.64
Medium	1.07 (0.70–1.66)	0.74								1.23 (0.76–1.98)	0.39	
High	0.74 (0.48–1.14)	0.17								1.00 (0.62–1.64)	0.98	
**Community level of men in higher education**												
Low	ref	ref	0.84							ref	ref	0.74
Medium	1.14 (0.73–1.80)	0.56								0.92 (0.56–1.51)	0.75	
High	1.07 (0.69–1.65)	0.76								1.12 (0.72–1.76)	0.61	
**Community level of men in cash employment**												
Low	ref	ref	0.03							ref	ref	0.03
Medium	0.69 (0.46–1.04)	0.08								0.67 (0.44–1.02)	0.06	
High	0.57 (0.37–0.88)	0.01								0.56 (0.35–0.89)	0.02	
**Community level of men who never justify IPV**												
Low	ref	ref	0.70							ref	ref	0.58
Medium	0.85 (0.52–1.41)	0.54								0.78 (0.47–1.29)	0.33	
High	1.02 (0.66–1.59)	0.92								0.95 (0.61–1.50)	0.85	
**Proportion of total residual variance explained by each level**												
Individual				3.29			3.29			3.29		
Community				0.95			0.93			0.73		
VPC^b^				0.22			0.22			0.18		
**Model fit indices**^c^												
AIC				1832.60			1628.09			1633.07		
BIC				1928.60			1760.07			1849.52		

a Figures derived from weighted sample of only 39 individuals.

b This is the variation partition coefficient. A VPC close to 0 suggests that little to no residual variation in IPV is attributable to variation among communities, so most of the variation is among individuals and thus there is little correlation among them. On the other hand, a VPC close to 1 suggests that most of the residual variation in IPV is attributable to variation among communities, so little variation is to be found among individuals; thus, there is high correlation among them.

c Based on simplified versions of the models not adjusting for clustering of the data. Lower values indicate better fit.

Model 2 included additional factors relating to a woman's relationship and household. The associations between a woman's age, education and experience of non-partner sexual violence, and her experience of IPV, reduced in size and became non-significant with the addition of the relationship and household characteristics into the model. The remaining individual characteristics (employment, parental IPV and experience of physical abuse from parents) remained significant in model 2, with similar effect sizes. With regard to the relationship and household characteristics, having a partner that drinks alcohol was strongly positively associated with experiencing IPV (OR 2.67; CI 1.93–3.70), as was experiencing any controlling behaviours from a current partner (OR 7.72; CI 3.88–15.36).

The final model, model 3, included additional factors relating to a woman's community. All factors that were significant in model 2 remained significant with the addition of the community characteristics, with very few changes in the size or strength of associations. There was a statistically significant protective association between living in a community with high levels of women with higher education (OR 0.49; CI 0.25–0.96), and medium levels of women involved in decision-making in their relationship (OR 0.62; CI 0.39–1.00). Likewise, living in a community with high levels of men in cash employment (OR 0.56; CI 0.35–0.89) was also significantly associated with a lower risk of IPV among women.

## Discussion

In this study, we show that factors across individual, relationship/household and community levels are associated with women's experiences of IPV in Samoa. These findings add to a growing body of evidence on the factors associated with women's experiences of IPV, particularly at the community-level and for the Pacific Islands region where there are significant gaps. We argue that community-level drivers are essential to understand for the development of community-based interventions. We also demonstrate that further research is needed to design tools to measure key community-level factors in order to understand how they contribute to high levels of IPV.

At the individual level, women's age and education were associated with experiencing IPV. Consistent with other studies across LMICs,^6^ younger women had greater risk of experiencing IPV, while more educated women had lower risk. These factors became statistically non-significant with the addition of relationship/household and community level factors. One factor that remained significant across all models was women's employment, in which taking part in both paid and unpaid work was associated with lower risk of experiencing IPV. The potential protective nature of paid employment is generally consistent across other studies in LMICs,^6^ suggesting that women who earn their own income and contribute financially to their family have greater bargaining power and status, as well as having the resources to leave violent relationships.^26^ This may be particularly relevant in the context of Samoa where in comparison to men, women are disproportionately engaged in the care economy (63.4% of women in this study were unemployed, the majority of whom reported that their main activity in the past seven days was domestic duties), which can be a contributing factor to women's socioeconomic disempowerment.^27^^,^^28^ On the contrary, there is also some evidence to suggest that employed women and those who earn a higher income than their partner are at increased risk of experiencing IPV due to the threat it can bring to masculinity and gender roles.^6^ Our finding that employed women are at lower risk of experiencing IPV in Samoa demonstrates the importance of developing deeper understandings of the socio-cultural context in order to unpack these complex relationships. Interestingly, women in Samoa who undertook unpaid work were also at lower risk of experiencing IPV. This is a novel finding and one that requires further exploration.

Exposure to violence in childhood was important in predicting IPV risk. This is consistent with many studies which demonstrate that IPV has an intergenerational nature, and those who witness and experience violence in childhood are more likely to be perpetrators or experience violence themselves in later life.^6^ A cross-sectional study in the Republic of Kiribati, another Pacific Island nation, found that experiencing physical abuse in childhood was a predictor of men's perpetration of IPV.^29^ In the Samoan context, there are widely held perceptions that the use of violence in the family is normal.^30^ Physical punishment of children by parents is used as an acceptable form of discipline and this perception is further reinforced by the use of corporal punishment in schools.^31^ The DHS-MICS found that 90.8% of children aged 1–14 years were subjected to at least one form of physical or psychological punishment by parents/caregivers in the last month.^23^ With high rates of physical punishment, IPV and children witnessing IPV in their families, a cycle of violence and its normalisation is perpetuated across generations.

Alcohol use is a well-known and widely evidenced factor associated with IPV perpetration and experience, understood to trigger or increase the severity of violent acts.^10^^,^^32^ Findings from this study were consistent, and women who reported their partner drinking alcohol were more likely to experience IPV. This finding was strong and consistent across all models. Alcohol use is considered to increase aggression through changes in cognitive functioning, ultimately increasing the perpetration of IPV through various pathways.^6^ Harmful alcohol use is a growing health and social issue in Samoa^30^ and is frequently referred to as a trigger of violence in the public discourse.^24^^,^^33^, ^34^, ^35^

We found three factors at the community level to be associated with women's IPV experience in Samoa: the community level of women with higher education, women involved in household decision making, and men in cash employment. Women who lived in communities with more women with higher education had a lower risk of experiencing IPV, consistent with a small number of studies in other LMICs.^18^^,^^21^ Living in a community with a higher level of women involved in household decision-making with their partner was also protective against women's individual IPV risk in our study. This is a novel contribution to the literature on community level protective factors for IPV in LMICs. Together, high levels of women's educational attainment and involvement in household decision-making at the community level possibly indicates greater levels of women's empowerment in a community. In the Samoan context, this might also be an indication of the strength of women's leadership in such communities. The presence of women in community leadership roles such as the *komiti* (village women's committees) and *mafutaga tina* (women's church groups) might translate into greater decision-making power for women within the household, and therefore reduced levels of violence, because of how women's opinions may be more valued in Samoa if they hold such roles. However, this finding must also be considered in light of the fact that, despite growing number of women holding leadership roles in Samoa in the past 30 years,^36^ religious beliefs, cultural values and social assumptions still produce barriers to women attaining decision-making power within traditional Samoan villages.^37^ Perceptions of men as leaders, fathers as the heads of families, and attitudes that women's roles are restricted to the domestic sphere limit women's opportunities for political participation at the local and national level.^37^^,^^38^ Moreover, given that our community level decision-making variable was derived from aggregating individual data on women's involvement in household decisions, our understanding of this pathway between levels of women's empowerment in the community and individual experiences of IPV is limited. This points towards the need for better tools that can measure factors at the community level, such as community level women's decision-making, to gain a more comprehensive understanding of how they influence violence experiences.

The final community level factor associated with women's experiences of IPV was the level of men's employment—women living in communities with higher male employment had a lower risk of experiencing IPV. While very few studies have explored this association, our findings complement those from a study in Tanzania which found that women living in communities with higher levels of unemployed men had an increased risk of experiencing past-year IPV.^12^ Authors hypothesised that male unemployment can increase IPV risk through increased household tension and conflict brought about by the consequences of joblessness. The mechanism through which community-level men's employment appears to act as a protective factor in Samoa could be similar. The burden of financial obligations in Samoan communities, such as regular contributions to the church, *taulaga,* has been linked to social and economic marginalisation including financial hardship and family violence in the household.^39^^,^^40^ One explanation behind a reduced risk of IPV among women living in communities with higher levels of employed men could be because the financial burden of *taulaga* is lessened in wealthier communities, possibly reducing household conflict and the stressors that lead to IPV. However, similar to women's decision-making at the community level, more research is needed to unpack this relationship further.

Our study has several limitations. The cross-sectional nature of the data limits our ability to draw conclusions about temporality and causality between exposure and outcome variables. We may also have missed other factors that could possibly be associated with women's IPV experience, such as women's exposure to media, which could be an avenue for future research. The aggregation of individual level data to derive community-level variables also limits our ability to understand the mechanisms through which dynamics at the community level influence individual IPV experiences. Including emotional violence in our IPV measure, as well as physical and sexual, is a strength of this study, enabling us to capture broader experiences of violence that we know to be important in this context^41^ beyond the widely used physical and/or sexual IPV measure. However, it is important to acknowledge that the standardised method for measuring experiences of IPV employed by the DHS methodology to create IPV variables has potential limitations, particularly in contexts where there is stigma associated with experiencing and reporting violence, such as Samoa, where experiencing violence can often be viewed as a shameful and private matter.^24^ Moreover, while standardised questions allow for comparability across countries and over time, such methods are limited in their ability to be ‘owned’ by local communities and can obscure local epistemologies of violence.^42^

The findings of our study have numerous implications for intervention development and research in Samoa and beyond. Given the importance of exposure to violence in childhood in predicting women's experiences of IPV, interventions in Samoa should take a whole family approach to violence prevention.^24^^,^^43^ Strategies would need to focus on reducing multiple types of violence, including IPV and child abuse, to end intergenerational cycles of family violence in Samoa. In this context, given that many families live in extended family arrangements, interventions would need to conceptualise family violence as something that extends beyond the household level to take a more holistic view of kin networks. As part of a multi-pronged approach, IPV prevention interventions should also target the intersections of gender, IPV, and problematic alcohol use, a factor that was strongly associated with IPV experience across all models in our study, and one that there is political will to tackle in this context. It is imperative to address triggers of violence, like problematic alcohol use and poor communication between couples, as components of larger programmes that address the structural drivers of IPV, such as gender inequalities.^44^ Gender transformative programmes, which aim to reshape gender relations to be more equitable, have potential in this context to support communities in critical reflection on how entrenched sociocultural norms can lead to IPV through perpetuating unequal perceptions about the roles of men and women in Samoan society and justifying the use of violence as a means of upholding these roles.^45^ Community-based interventions in Samoa should also focus on women's empowerment at the community level as part of a structural approach, including interventions that support women's participation in village leadership and decision-making roles. At the individual and community-level, these spaces create channels through which women can seek support for experiences of violence, as well as a sense of belonging, value and status within the community. Beyond this, women's participation in local leadership and governance paves the way for more equitable national political participation, which is crucial for achieving women's empowerment and gender equality in Samoa, the Pacific region, and globally.^46^ Examples of such approaches across the Pacific include the Women in Leadership in Samoa joint initiative by UN Women and UNDP aimed at training community members in developing leadership skills.^38^^,^^47^ However, more evidence is required to understand the effectiveness of these structural approaches in reducing VAWG.

Our findings highlight that conducting community-level analyses provides critical insight into community dynamics that are essential when developing and implementing community-based interventions. Rather than transferring approaches from one context to another, community-based interventions should build on existing mechanisms through which violence is responded to and prevented locally.^48^ They should also draw upon findings from community-level analyses which highlight factors that may be protective against IPV, such as community levels of men's employment, women's education and women's decision-making, as found in this study. Findings from this study also have implications for future research. Thus far, most studies exploring community-level drivers of IPV have used aggregated individual level data to create community-level variables. This approach is limited and our analysis points towards a need to design tools to specifically measure variables at the community level that allow us to explore relationships, interactions and behaviours that emerge as a product of community dynamics. This work could draw on emerging approaches that have measured social norms^49^ and collective action norms^50^ within community settings to unpick how community dynamics can lead to, or be protective against, violence. With a better understanding of how community contexts drive IPV in individual relationships, community-based interventions can be better informed to create environments at the community level that support meaningful and sustainable change towards VAWG prevention.

## Contributors

HL, JM and LB conceptualised the study. HL conducted data analysis with supervision from JM and LB. TF, LS and LT provided support with data interpretation. All authors reviewed and edited the manuscript before providing final approval for submission.

## Author reflexivity statement

The authors on this paper have expertise in areas including gender-based violence, intervention co-development and implementation, and the analysis and implementation of population-based surveys. Our work is informed by feminist and decolonial perspectives that see violence against women and gender-based violence as driven by inequitable societal structures. Two authors are based in the UK and four in Samoa. Four of the authors work at higher education institutions (UK and Samoa), with varying levels of seniority, including early-career researchers, and two authors work at the Samoa Bureau of Statistics as statisticians and in leadership roles.

## Data sharing statement

The dataset used in this manuscript is available from: https://mics.unicef.org/.

## Research ethics approval

The survey protocol for the DHS-MICS data collection was approved by Samoa DHS-MICS Steering Committee. Secondary analysis of this dataset did not require ethical approval.

## Declaration of interests

The authors declare no competing interests.

## Footnotes

d The World Health Organisation Violence Against Women prevalence estimates data published in the Lancet estimated the prevalence of lifetime physical, sexual and/or emotional IPV to be 40% among women aged 15–49 in Samoa. This figure was derived from pooling data from all nationally representative population-based surveys from 2000 to 2018.^1^
